# IMPLICATIONS OF RESIDENT AND ENVIRONMENTAL CHARACTERISTICS ON RESIDENT-TO-RESIDENT AGGRESSION

**DOI:** 10.1093/geroni/igad104.0148

**Published:** 2023-12-21

**Authors:** Elsie Yan, Daniel W L Lai, Sheung Tak Cheng, Timothy Kwok, Vivian Weiqun Lou, Habib Chaudhury, Karl Pillemer, Mark Lachs

**Affiliations:** THe Hong Kong Polytechnic University, Hong Kong, Hong Kong; Hong Kong Baptist University, Hong Kong, Hong Kong; The Education University of Hong Kong, Hong Kong, Hong Kong; The Chinese University of Hong Kong, Shatin, Hong Kong; THe Hong Kong Polytechnic University, Hong Kong, Hong Kong; Simon Fraser University, Vancouver, British Columbia, Canada; Cornell University, Ithaca, New York, United States; Weill Cornell Medicine, New York City, New York, United States

## Abstract

This study examines factors associated with resident-to-resident aggression (RRA) in long term care facilities in Hong Kong. A total of 800 personal care worker participated. Participants averaged 42.03 years of age (SD=7.63), were mostly female (92.7%), married (79.1%) and reported an average of 6.28 years of experience in long term care. 96.9 percent of the participants provided care to residents with dementia but 58.9% considered the training they received insufficient. RRA was common: All participants reported having witnessed verbal aggression (100%), 18% disruptive behaviors, 11.8% physical violence, and 3.1% sexual aggression. The following analysis used a subset of 412 participant from 29 long term care facilities where environmental assessments were conducted. General linear model analysis showed that RRA was associated with both resident and environmental characteristics. Victims’ neuropsychiatry symptoms, perpetrator male gender and neuropsychiatric symptoms were associated with higher RRA. Separation of residents with and without dementia, inappropriate use of nursing unit for residents as measured by Therapeutic Environment Screening Survey for Nursing Homes (TESS-NH), lack of functional and attractiveness courtyard, less cleanliness of spaces, insufficient lighting and insufficient orientation / cueing as measured by Professional Environmental Assessment Procedure (PEAP) were also associated with higher RRA. There is an urgent need to prevent and intervene RRA in long term care facilities. Improving management of neuropsychiatric symptoms and creating a supportive and accommodating environment could be helpful in this regard.

